# Down but Not Out: The Consequences of Pretangle Tau in the Locus Coeruleus

**DOI:** 10.1155/2017/7829507

**Published:** 2017-09-05

**Authors:** Termpanit Chalermpalanupap, David Weinshenker, Jacki M. Rorabaugh

**Affiliations:** Department of Human Genetics, Emory University School of Medicine, Atlanta, GA 30322, USA

## Abstract

Degeneration of locus coeruleus (LC) is an underappreciated hallmark of Alzheimer's disease (AD). The LC is the main source of norepinephrine (NE) in the forebrain, and its degeneration is highly correlated with cognitive impairment and amyloid-beta (A*β*) and tangle pathology. Hyperphosphorylated tau in the LC is among the first detectable AD-like neuropathology in the brain, and while the LC/NE system impacts multiple aspects of AD (e.g., cognition, neuropathology, and neuroinflammation), the functional consequences of hyperphosphorylated tau accrual on LC neurons are not known. Recent evidence suggests that LC neurons accumulate aberrant tau species for decades before frank LC cell body degeneration occurs in AD, suggesting that a therapeutic window exists. In this review, we combine the literature on how pathogenic tau affects forebrain neurons with the known properties and degeneration patterns of LC neurons to synthesize hypotheses on hyperphosphorylated tau-induced dysfunction of LC neurons and the prion-like spread of pretangle tau from the LC to the forebrain. We also propose novel experiments using both *in vitro* and *in vivo* models to address the many questions surrounding the impact of hyperphosphorylated tau on LC neurons in AD and its role in disease progression.

## 1. Introduction

Alzheimer's disease (AD) is an insidious and debilitating illness affecting approximately 46.8 million people worldwide [1], representing a significant social and economic burden. AD has proven difficult to study and treat due to the combination of a long prodromal phase and the heterogeneous nature of clinical symptoms and pathology. Despite intensive research examining the pathologic hallmarks of AD, amyloid-*β* (A*β*) plaques and tau neurofibrillary tangles (NFT), no disease modifying therapies have emerged [2]. Clinical trials targeting mid- to late-stage AD have failed, potentially due to the irreversible neuronal damage and loss that has already occurred by the time of diagnosis. As a result, research efforts have shifted towards earlier AD detection and treatment.

One area of interest in early AD involves the locus coeruleus (LC), the major brainstem noradrenergic nucleus that innervates and supplies norepinephrine (NE) to the forebrain [3]. In search of the earliest detectable AD-like neuropathology, Braak and colleagues surveyed postmortem brains from over 2000 individuals throughout the lifespan for A*β* and aberrant forms of tau and found evidence that hyperphosphorylated tau began accumulating in the LC of individuals around 40 years of age [4]. Notably, pretangle tau in the LC was *always* detected in brains containing A*β* plaques but was also found on its own in brains from younger individuals in the absence of all other AD-related pathologies. Based on these results, Braak amended his canonical staging of AD to include the LC as the first brain region to show tau pathology [4, 5]. These studies had several limitations and were initially questioned. For example, because postmortem analysis was employed, it is impossible to know whether the individuals with hyperphosphorylated tau in the LC would have eventually developed AD. Furthermore, Braak examined early forms of hyperphosphorylated tau, and initial studies failed to find more advanced forms of hyperphosphorylated tau or NFTs reminiscent of AD pathology in the forebrain [6]. However, recent studies have confirmed a variety of pretangle tau species and NTFs in the LC that increase with AD severity [7, 8]. Finally, hyperphosphorylated tau staining in early AD has been reported in the dorsal raphe nucleus (DRN), which together with the LC forms part of the isodendritic core (IC) that appears particularly vulnerable to neurodegeneration [8, 9]. Thus, while the exact origin of aberrant tau in AD is still under debate, it is clear that hyperphosphorylated tau in the LC is among the first detectable signs of AD-like neuropathology in the brain. The detrimental role of hyperphosphorylated tau (both due to loss of normal function as well as gain of toxicity) has been heavily investigated, but mainly in the context of cortical neuron populations (reviewed in [10, 11]). Although LC neurons die in later stage disease (see below), it is not known whether pretangle tau pathology precipitates their degeneration.

The LC is a small nucleus (~22,000–55,000 and 1500 neurons per hemisphere in humans and rodents, resp.) in the brainstem and the main source of NE for the forebrain [3]. Despite the size of the nucleus, LC neurons innervate most of the brain, excluding the basal ganglia [12–15]. For the purposes of this review, we will highlight key findings relevant to AD but direct the reader to several excellent sources for a comprehensive description of LC neuroanatomy [3, 16, 17]. LC neurons send collateralized projections throughout the brain from topographically distinct regions along the rostral/caudal and dorsal/ventral axes [15], which exhibit projection-specific geometry and vulnerability in AD. In early AD, pretangle tau accumulation is the greatest in the middle third of the LC, which densely innervates the hippocampus and cortex [8, 15, 18]. As AD progresses, cell loss is concentrated to the rostral and middle sections of the LC, leaving the caudal potion, which primarily targets the cerebellum, spinal cord, and other brainstem nuclei, relatively spared [15, 19]. The cause of this apparent selective regional vulnerability is unknown but could be the result of excitotoxic glutamatergic inputs from the cortex, which are concentrated in the middle of the LC [3]. In addition, the dorsal portion of the LC projects heavily to AD-associated regions such as the entorhinal cortex (EC), hippocampus, and frontal cortex, while the ventral portion innervates the spinal cord, potentially indicating specialized function [15, 18]. Indeed, recent work has shown that LC neurons have unique cellular properties based on their projection targets [20]. Combined, these results suggest that distinct populations of LC neurons may be especially vulnerable to degeneration based on projection targets and cellular properties.

Canonical roles for the LC have been described for attention, stress responses, and arousal; these studies have been extensively reviewed and will not be covered in detail here [16, 17, 21]). Degeneration of the LC is a ubiquitous feature of AD [19, 22–24], occurring in mid- to late- stage disease (~Braak III-IV)[19, 25]. Moreover, loss of LC neurons better predicts onset and severity of AD symptoms and A*β*/NFT pathology than cell loss in any other brain region implicated in AD, including the hippocampus, EC, and nucleus basalis of Meynert [26]. NE has anti-inflammatory properties, primarily mediated via β-adrenergic receptors (ARs) on glial cells, and directly enhances A*β* clearance via microglia activation [27]. Besides NE, LC neurons also produce and release brain-derived neurotropic factor (BDNF), a potent trophic peptide that enhances synaptic plasticity and A*β* clearance while reducing tau phosphorylation [28]. BDNF is particularly important for maintaining LC innervation in forebrain in old, but not young, rats and LC-derived BDNF may represent a resilience factor for age-dependent cell loss in AD [29]. Thus, it is not surprising that chemical or genetic elimination of LC neurons enhances cognitive deficits, cortical inflammation, and A*β* deposition in amyloid-based mouse models of AD [30–36]. Although LC lesioning studies have not been reported in transgenic tau models, LC lesions in APP transgenic mice increase forebrain hyperphosphorylated tau levels, suggesting that LC degeneration may impact tau pathology [37]. While losing LC neurons may be a turning point in AD progression, it appears that pretangle tau accumulates in LC neurons decades prior to their demise [4, 19, 25]. Since hyperphosphorylated tau is known to disrupt cellular function, this begs the question of whether aberrant tau accumulation is altering LC neuron function in mild cognitive impairment (MCI), a prodromal form of AD, and early AD. This review focuses on the potential impact of hyperphosphorylated tau accrual on LC neurons and suggests experimental approaches to probe the functional relationship between hyperphosphorylated tau and the LC in AD progression.

## 2. Role of Hyperphosphorylated Tau on LC Morphology and Function

Pretangle tau emerges in the LC in individuals around 40 years old, yet degeneration of LC neurons is not apparent until mid-stage AD, suggesting that LC neurons could be harboring pathogenic tau species throughout the decades-long prodromal stage of AD. Given that hyperphosphorylated tau in cortical neurons disrupts a wide variety of functions, it seems likely that it is also corrupting LC function, perhaps early in AD.

Since LC degeneration was first described in AD, it was also noted that surviving LC neurons are morphologically ragged, with “swollen and misshapen” somas, reduced arborization, and thick, shortened neurites [38]. Alterations in LC innervation have been reported in the hippocampus and cortex of AD patients. Morphologic changes in LC fibers along with decreased LC fiber density [38, 39], tissue NE levels [40], and compensatory increases in ARs [41] indicate noradrenergic dysfunction and potential retraction of LC innervation from these regions in AD. Whether hyperphosphorylated tau accumulation contributes to this dysfunction is not known. One study found increases in hippocampal *α*2AR levels, a compensatory sign of low NE transmission, in Braak I, a stage when tau pathology begins to appear in the EC [7]. In the forebrain, hyperphosphorylation of tau leads to its dissociation from microtubules and causes disassembly, impairing axonal stability, axonal transport, and neurotransmission [42, 43]. Likewise, LC neurons from MCI and AD patients, which have increased expression of the 3R:4R tau isoforms and decreased expression of a variety of axonal and synaptic structure proteins, a pattern consistent with structural instability [25]. Notably, mRNA expression of *map1b* and *synaptopodin*, which are negatively correlated with global cognition scores across AD development, suggest a link between instability and cognitive decline [25]. A recent study found that LC neurons containing hyperphosphorylated tau have fewer synaptic inputs than neighboring LC neurons without pathogenic tau, indicating that hyperphosphorylated tau may contribute to synapse retraction [7]. Moreover, inflammatory markers are upregulated in LC neurons across Braak stages I–IV, suggesting that hyperphosphorylated tau may also produce local inflammation [7]. While it is not yet clear what causes LC dysfunction and degeneration, the cross-sectional studies allude to the potential role of aberrant tau.

LC dysfunction and degeneration have been identified in a variety of neurodegenerative diseases including AD, Parkinson's disease, chronic traumatic encephalopathy, and multiple sclerosis that impact the aforementioned IC [44–47]. The IC is a conserved set of nuclei, containing the LC, DRN, substantia nigra, and basal nucleus of Mynert that share many cellular and anatomic features and are similarly affected in AD [9]. Dysfunction in the IC, especially the LC and DRN, has been postulated to contribute to disruptions in mood and sleep that precede memory loss in AD [9]. New stereological experiments have detected pretangle tau in the DRN as well as the LC in early AD (Braak 0) [19]. In addition, nuclei in the IC release neuromodulators via volume transmission rather than specialized synaptic contacts. Due to this mode of signaling, disruptions in these neuromodulators are likely to have a broad impact on wide populations of neurons and glia compared to synaptic transmission. The neurons in the IC share fundamental properties including long, unmyelinated projections that create high bioenergetic demand and may make these neurons particularly sensitive to a variety of insults [3, 48, 49]. Similar to substantia nigra neurons, LC cells have tonic firing patterns due to calcium influx from L-type calcium channels [50], and one study found that tonic activity and L-type calcium channels increase mitochondrial stress in LC neurons, leading to cellular dysfunction over time [50]. Similarly, cognitive performance in amnestic MCI and AD patients is negatively correlated with disruption of mitochondrial and cellular stress proteins in LC neurons, suggesting that early cellular stress in LC neurons may be contributing to AD progression [25]. Furthermore, the LC is a critical mediator of the physiologic stress response, and chronic stress increases tau hyperphosphorylation in the LC of mice [51]. Taken together, LC neurons appear to be susceptible to both cellular and physiologic stressors.

The LC is also anatomically susceptible due to its position near the fourth ventricle and significant vascularization by blood vessels devoid of the blood brain barrier (BBB) [52]. Thus, LC neurons are likely exposed to high levels of toxins, pathogens, and peripheral immune cells lurking in the cerebrospinal fluid and blood [44, 49]. In fact, heavy metals, presumably from the blood, can be detected in the LC, although metal-containing neurons seem to be distinct from those that contain hyperphosphorylated tau, suggesting some regional/cell type specificity for pathology in the LC [52]. Furthermore, chemically lesioning the LC disrupts the BBB; dysfunction/degeneration of the LC may impact neuroinflammation throughout the brain and contribute to the leaky BBB observed in AD patients [31, 53].

Neuroinflammation, which is profoundly influenced by the LC/NE system, is a hallmark of AD (reviewed here [27, 54]). A proinflammatory environment in the forebrain is an early phenotype of many mouse AD and tauopathy models, yet few have examined whether inflammation occurs in the LC. Recent evidence indicates the increasing presence of microglia and expression of interleukin 6 in the LC across the Braak stages in humans [7]. Similarly, rats expressing wild-type human tau have increased expression of inflammatory cytokines and reductions of the NE biosynthetic enzyme, tyrosine hydroxylase, in the LC, and hippocampus [55]. Chronic infusion of lipopolysaccharide into the 4th ventricle resulted in exposure- and age-dependent increases in microglia and concurrent declines in LC neuron density [56]. Combined, these data suggest that hyperphosphorylated tau and inflammation may reduce LC neuron health, especially with age. Due to the interconnected nature of inflammation, tau pathology and LC/NE signaling, careful examination of the interplay between these factors is warranted.

While hyperphosphorylated tau is likely disrupting LC function, whether hyperphosphorylated tau is itself toxic or rather confers sensitivity to secondary insults (i.e., a “two-hit” model), it is still unresolved [10, 11]. Postmortem evidence suggests that if pretangle tau toxicity exists in the LC, it is not swift and takes years or even decades to result in neuron loss [4, 19]. Given the timing, the two-hit model seems an attractive hypothesis because the LC seems anatomically destined to experience chronic toxicant exposure from the blood and CSF [49]. One possibility is that LC neurons may be somewhat resilient to stressors early in life but succumb to them overtime. If this is correct, identifying factors contributing to resilience and extend them could delay onset or progression of a variety of neurodegenerative diseases. Yet, no mechanistic studies assessing the effect of pretangle tau on LC neuron survival have been published, and additional research is necessary to answer these questions. In sum, the structure of LC neurons in combination with their metabolic demands and anatomic position confers both increased exposure and sensitivity to cellular stressors. In light of these characteristics, it is perhaps not surprising that LC neuron dysfunction and degeneration are implicated in a variety of neurodegenerative diseases in addition to AD. Further study of LC neurons is necessary to understand their cellular vulnerability and resilience, especially in the context of hyperphosphorylated tau.

## 3. *In Vitro* Model Systems to Examine the Consequences of Hyperphosphorylated Tau in LC Neurons

The techniques available to examine LC neuron function *in vitro* with cell-type specificity are limited due to the small size of this nucleus that requires fine dissection and low tissue yields for traditional approaches. While hyperphosphorylated tau disrupts normal cellular function and leads to abnormal morphology and neurotransmission in hippocampus neurons *in vitro* and in transgenic mice [10, 57, 58], little attention has been paid to the LC. One approach could be the use of immortalized catecholaminergic cell lines and primary LC cell cultures expressing various forms of wild-type and pathogenic tau to examine the impact on LC neuron morphology and neurotransmission via microscopy and biochemical methods already validated for forebrain neurons. Dissection for primary cultures could be aided by the use of TH-GFP mice and rats, which creates fluorescent catecholamine neurons and has been employed to facilitate making dopamine neuron cultures [59]. Furthermore, these cultures could be used to examine the sensitivity of LC neurons to a variety of stressors (toxins, immune stress, tau fibrils, etc.). By utilizing TH-GFP rodents to visualize living LC neurons, these results could also be translated to slices to assess real-time imaging of immune infiltration and tau engulfment, as well as electrophysiological recordings to examine the effect of hyperphosphorylated tau on neuron activity. The ability to visualize and selectively manipulate signaling cascades makes these *in vitro* and *ex vivo* systems extremely powerful and well equipped to examine how hyperphosphorylated tau affects LC neuron health in the context of AD-related pathology and stressors in the LC microenvironment. *In vitro* systems are also ideal for high-throughput screening of small molecules that modulate hyperphosphorylated tau accrual and degeneration in the LC.

## 4. Tau Seeding and Spread from the LC

In AD, tau pathology is first detected in the LC, then appears in the interconnected brain regions in a temporarily and spatially distinct manner [4]. Some of the densest LC projections can be found in the EC and dentate gyrus of the hippocampus, the next brain regions affected in Braak I and II, respectively, before eventually reaching other parts of the forebrain (e.g., cerebral cortex) [3, 5]. This pattern mirrors the clinical symptoms, beginning with early changes in a variety of noncognitive areas like sleep and affect, attributed to alterations in the IC, followed by subtle memory impairment, and the steady decline across higher function cognitive domains [9, 60]. This distribution has driven the hypothesis that pathogenic tau can spread transsynaptically *in vivo* via “templated protein corruption” or “seeding” [61]. Indeed, injections of mouse mutant tau aggregates [62, 63], human AD brain lysate [64, 65], recombinant tau [66], or tau-expressing virus [42] into the forebrain of mice induces tau pathology near the injection site as well as in the interconnected brain regions, indicating spread. Furthermore, the spread of early tau pathology has been observed in mice that express aggregate-prone tau only in the EC, an area affected in Braak I [43, 67]. While the precise mechanism of how intercellular tau protein propagates remains unclear, proposed mechanisms include extracellular release and reuptake, possibly in conjunction with neuronal activity or firing [58]. Notably, neuronal activity hastens the spread of tau pathology from the EC to the DG, and A*β* pathology from the hippocampus to the forebrain, suggesting that dampening neuronal activity may retard pathogenic protein spread in AD [68, 69].

There is much debate on whether the pretangle tau in the LC indicates that the LC truly initiates AD tau pathology. While studies show the presence of pretangle tau in the LC in young individuals, there is no way to determine whether these individuals would have eventually developed AD. Other factors (repeated concussions, stress, and heavy drug use) also increase hyperphosphorylated tau in the LC [46, 51, 55, 70] and could trigger the appearance of aberrant tau. The only attempt to examine the spread of tau pathology from the LC experimentally delivered puzzling results. Transgenic mice expressing aggregate-prone human tau that were given intra-LC injection of preformed tau fibrils showed pretangle tau spread to many areas of the brain, but oddly spared the expected AD-associated targets, the EC, and hippocampus [71]. Because the results did not recapitulate the stereotyped pattern of tau pathology in early AD (LC-EC-hippocampus), the authors suggested that pathogenic tau does not spread from the LC in human disease; this interpretation may be premature, and study limitations should be considered. For instance, although fibril injections are a widely used model of prion-like protein spread, attention should be given to the source of tau and brain region. First, various tau species are differentially taken up and transported in neurons depending on their size, isoform, and modifications [72–74]. Thus, it is plausible that the conformation/species of the synthetic tau fibrils injected into the LC were preferentially targeted to the brain regions other than the EC/hippocampus. Moreover, the PS19 mice and fibrils used in this study express a P301S mutant tau, which has different seeding capacity than tau aggregates in AD [75]. Second, because catecholaminergic and glutamatergic neurons have very different cellular properties, the mechanisms underlying tau internalization and spread may also differ. Furthermore, LC neurons are not homogeneous, and it is possible that differences in electrophysiological properties may favor internalization and spread to the forebrain but not the EC or hippocampus [20]. One potential solution to these limitations is to develop a model that selectively expresses wild-type human tau in the LC and therefore has a wider compliment of tau species, does not rely on tau internalization, and may more representative of tau spread from this region in humans. Whether the LC represents the initial site of pathology or reflects a nonspecific response to brain insults is still under debate [6]. Additional studies, using a variety of seeding models, are necessary to resolve this question.

Regardless of the model system used, the field could benefit from more investigations focused on the age-dependent emergence of tau pathology across brain regions. For example, while Braak staging is the gold standard from a clinical standpoint, it is rarely applicable in animal models because tau transgenes are typically driven by ubiquitous promoters that express tau in all neurons simultaneously. As the AD field pivots to early disease detection and treatment, examination of early pathology, especially within the LC, of animal models may present new models for understanding early disease mechanisms and potential therapies.

## 5. *In Vivo* Models of Tau Spread

The majority of tau propagation experiments have been performed in mice. Compared to humans, mice express fewer tau isoforms, and those that are expressed are resistant to hyperphosphorylation and aggregation [76]. Because it is very difficult to coax endogenous mouse tau to form aberrant species, tau seeding studies have generally been done in transgenic mice that ubiquitously express mutant tau [57]. While this provides a receptive template for tau propagation, mutant tau has different properties than wild-type tau, and the relevance of this model to AD is unclear [72–74]. In fact, unphosphorylated, rather than hyperphosphorylated or aggregated, tau was found to be the agent of spread in rats with virally expressed wild-type human tau in the hippocampus [42]. In addition, obvious confounds exist when using ubiquitous expression of tau to study spread between brain regions. Rats present a potential solution to these issues. Rats express the same 6 isoforms of tau as humans, albeit in a different 3R : 4R ratio (1 : 9 in rats and 1 : 1 in adult humans) [76]. Nevertheless, rats reproduce some aspects of tauopathies that mice do not [76–78]. Notably, conversion of wild-type human tau into pretangle tau occurs in SHR72 rats expressing the 4R human tau isoform, but not mice harboring a similar transgene [78, 79]. In addition, tau pathology propagates from the hippocampus in rats injected with SHR72 rat homogenate [78] or viral-mediated expression of human tau [42]. The power of using rats for tau research is perhaps best characterized by the TgF344-AD rat, which harbors the same mutant amyloid precursor protein and presenilin 1 transgene used to make the APP/PS1 mice [77, 80]. This transgenic rat is, to our knowledge, the only animal model that manifests *endogenous* tau pathology, while its APP/PS1 mouse counterpart does not [76, 77]. While in the past, mice were an economically and technically favorable species for genetic models, additional consideration should be given to rat models, especially given recent advances in gene editing technology. Where appropriate, rat-based models may be able to recapitulate missing pieces of the tau puzzle, including conversion of wild-type human tau to pathogenic species, the generation of endogenous tauopathy in response to amyloid and the propagation of tau in the absence of widespread mutant tau expression.

While tau spread in the hippocampus and cortex has been examined under a variety of conditions [42, 62–66], only the aforementioned single study using fibril injections has been examined in regards to seeding from the LC [71]. Exclusive anatomic targeting of tau fibrils to the LC is technically very challenging due to the small size of this nucleus (1500 neurons in a rodent), and there is no way to ensure noradrenergic neuron specificity. By contrast, genetic strategies have been successful in targeting LC neurons to produce effector and reporter proteins with cell-type specificity through promoter-driven expression using NE synthesis enzymes (TH, DBH) [81–83], LC transcription factors (Phox2b) [84, 85], and LC specification genes (Ear2 and DBH/En1) [36, 86, 87]. Recent work has shown selective and robust viral-mediated expression using Phox2b-dependent promoters [84, 85], Cre-dependent expression in TH-Cre mice [81, 88], or microinjections of virus containing a constitutive promoter [89]. This viral-mediated approach has several advantages for answering LC-based questions in a wide variety of existing AD models. By using intra-LC infusion of virus and an LC-specific promoter, one can achieve expression of desired proteins exclusively in LC neurons without the time and cost of traditional transgenic methods that often produce expression in other noradrenergic cell populations [84]. This approach is especially well suited for the investigation of tau seeding from the LC, where a variety of tau mutants could be expressed in the LC of a mouse or rat and examined for spread over time. This technique would allow for a high degree of anatomic specificity as well as a continuous, cell-autonomous source of tau unachievable by tau fibril infusion. Moreover, this approach is compatible with the latest brain clearing techniques (CLARITY, 3DISCO, and SeeDB) that would allow for more thorough investigation of tau spread [90–92].

## 6. Modulation of Tau Spread by LC Activity

Hippocampal hyperexcitability occurs in humans with MCI and rodent models of tauopathies and AD [93–97]. In mice, AD-associated hyperexcitability is reversed by tau deletion, suggesting that tau is the causative agent [95, 96, 98]. Synaptic activity facilitates tau transfer *in vitro*, and chemogenetic activation of EC neurons enhances tau spread *in vivo* [68, 95, 98]. These results are consistent with a feed-forward mechanism in which pretangle tau accrual promotes hyperexcitability, in turn facilitating its release and spread. Similar to its effects on hippocampal neurons, hyperphosphorylated tau may enhance LC neuron firing and facilitating tau spread from this nucleus. If true, this would imply that LC hyperactivity may facilitate AD progression, which at face value would be contrary to clinical evidence indicating that LC *degeneration* worsens AD. These results would not necessarily be mutually exclusive and may be dependent on disease stage. For example, enhanced LC activity early in AD, prior to cell loss or substantial drops in NE neurotransmission, may be harmful due to the enhanced spread of tau. By contrast, driving LC activity later in AD progression when the LC is degenerating, NE levels are low, and tau pathology is already abundant in the forebrain which may alleviate cognitive symptoms and retard furthering disease progression. Indeed, restoring NE levels with L-DOPS, a synthetic NE precursor, reduces A*β* pathology and/or improves memory in 5xFAD and APP/PS1 mice [31, 32]. Likewise, DREADD-induced LC activity restored behavioral performance in a mouse model of Down syndrome, which shares many APP-related pathologies [85]. Additional research examining potential tau-mediated LC hyperactivity, as well as activity-dependent tau spread from these neurons, is necessary to determine this relationship.

Whether hyperactivity in LC neurons contributes to AD pathogenesis is further complicated by the firing patterns of LC neurons. Similar to other catecholamine neurons, LC cells fire in two distinct modes: low tonic (sustained 0.1–5.0 Hz) and high phasic (10–20 Hz bursts) [21]. LC function follows the Yerkes-Dodson curve, with low tonic firing being associated with inattentive behavior, while high tonic firing leads to distractibility and is aversive in mice [21, 81]. Tonic activity is important for behavioral flexibility, while phasic activity is required for attention and optimal task performance [21]. Thus, if pretangle tau were causing LC “hyperactivity,” alterations in tonic or phasic firing could produce opposing effects on valence and cognition. It is also possible that the enhancement of tau spread is specific to a particular firing pattern. For example, neuropeptides produced by LC neurons such as galanin are thought to be preferentially released during phasic bursting [99–101]; this may also be true for tau. Activity-dependent tau spread has only been examined in excitatory neurons, which show very little tonic activity, and thus it is difficult to hypothesize whether tau transmission occurs selectively in either firing state. However, enhanced tau propagation is observed following either DREADD-induced tonic firing or optogenetic-induced burst firing, suggesting no firing state preference for tau transmission [68]. Whether this holds true for LC neurons will need to be investigated.

Whether hyperphosphorylated tau directly causes LC hyperactivity could be examined by electrophysiology in *ex vivo* slice preparations or *in vivo* recordings, and therapies that modulate LC excitability could be developed. If hyperactivity is enhancing tau spread, then reducing LC activity should retard propagation. This could be achieved using optogenetics, chemogenetics, or pharmacological agents. For example, the NE transporter inhibitor atomoxetine suppresses tonic LC activity while leaving phasic bursting largely intact, which could have the advantage of inhibiting tau spread while preserving cognition [102]. In future studies, it will be important to examine and manipulate LC firing to gain further understanding of early disease states and potentially elucidate novel avenues for AD treatment.

## 7. Conclusion

The exact role of hyperphosphorylated tau in the LC in the context of AD is shrouded in mystery. However, the ever-mounting clinical data suggests that at the very least, the LC contributes to disease progression and may be the site of its earliest manifestations. It is in our best interest to explore a variety of circuits and neuromodulators impacted in AD and not just the canonical brain regions and signaling cascades. The LC system represents an especially attractive candidate, since these neurons project to and modulate key nuclei affected in AD, and NE has powerful influences on cognition and behavior. In addition, it is somewhat remarkable that LC neurons appear to be able to tolerate aberrant tau species for many years before succumbing to frank degeneration, indicating that a large therapeutic window of opportunity exists when LC neurons are dysfunctional but not dead. We propose that future research focuses on two questions: (1) what is the impact of hyperphosphorylated tau on LC function and (2) can/does LC-derived tau seed forebrain pathology. The answers to these questions will inform the development of LC-based therapies for the prevention and/or treatment of AD.
